# Molecular and Structural Characterization of MHC Class II β Genes Reveals High Diversity in the Cold-Adapted Icefish *Chionodraco hamatus*

**DOI:** 10.1038/s41598-019-42003-5

**Published:** 2019-04-02

**Authors:** Marco Gerdol, Daniela Lucente, Francesco Buonocore, Elia Poerio, Giuseppe Scapigliati, Simonetta Mattiucci, Alberto Pallavicini, Roberta Cimmaruta

**Affiliations:** 1 Department of Life Sciences, University of Trieste, Trieste, Italy; 2 University of Tuscia, Viterbo, Italy; 3 Department for Innovation in Biological, Agrofood and Forest Systems, University of Tuscia, Viterbo, Italy; 4 Department of Public Health and Infectious Diseases, Section of Parasitology, Sapienza University of Rome, Rome, Italy; 5 Department of Ecological and Biological Sciences, University of Tuscia, Viterbo, Italy; 6 National Institute of Oceanography and Applied Geophysics, Borgo Grotta Gigante 42/C, Sgonico (TS), 34010 Italy

## Abstract

This study reports the presence of two distinct MHC class II β genes in the Antarctic icefish *Chionodraco hamatus*, belonging to the classical (ChhaDAB) and nonclassical (ChhaDBB) evolutionary lineages. By the application of targeted sequencing approach, a remarkable molecular diversity in the exon 2 sequence of the highly expressed gene ChhaDAB has been observed, resulting in an estimate of 92 different variants translated in 87 different peptides from 54 analysed icefish individuals. A highly conservative estimate, based on a 95% sequence identity threshold clustering, translate this variability in 41 different peptide clusters belonging to four different clades and showing the signature of different kinds of selection. In stark contrast, the poorly expressed ChhaDBB gene displayed a very low level of molecular diversity within exon 2, in agreement with expectations for a nonclassical MHC class II β gene.

## Introduction

Due to their key role as components of the adaptive immune system, the major histocompatibility complex class I and II molecules (MHC I and MHC II) have been widely studied in vertebrates^1–3^. These molecules are heterodimers formed by two subunits (α and β), each composed by two domains, which bind self and non-self-peptides and present them to T-cells. MHC II molecules primarily reside on the surface of antigen-presenting cells and exert their action interacting with CD4+ T helper cells. The MHC genes are the most polymorphic in vertebrates, with over 1000 alleles recorded in humans, and so enabling the binding of a large number of different peptides^4,5^.

One of the major discoveries in the study of MHC class I and II in teleost species resides in the different structural organization of their genes compared to other vertebrate groups^6,7^. While tetrapods (including mammals) and cartilaginous fish share highly organized MHC I and MHC II loci residing on the same linkage group, teleosts display an unlinked organization, as proved in a number of species belonging to both old and modern lineages^8^. Moreover, the number of functional copies of MHC II β genes can vary across species, ranging from one, as in salmonids and in the seahorse *Hippocampus abdominalis*, to more than ten copies, as in cichlids^9–11^. Finally, within the same species, the total number of MHC class II β loci can vary both between distinct populations and among single individuals belonging to the same population^12^. The overall picture is further complicated by the growing body of evidence that supports the co-existence of multiple evolutionary lineages of MHC II genes in a given teleost species^13–15^. In fact, comparative phylogenetic studies have highlighted that these sequences fall into distinct lineages that can be classified according to a classical or nonclassical definition^7,14^. A typical classical MHC class II β sequence is characterized by high expression and polymorphism, and by the conservation of key residues involved in the formation of hydrogen bonds with the backbone of peptide ligands and in the binding of CD4 (A lineage). On the other hand, the nonclassical sequences show low polymorphism, different expression patterns compared to classical counterparts and/or lack of binding capacity of CD4 (B lineage)^7,16^. Until now, nonclassical sequences have been identified in some teleosts like medaka, salmonids, tilapia, zebrafish and stickleback^15–17^, but their low levels of polymorphism and tissue-specific nature still leave unresolved questions on their biological functions. A third lineage (the E lineage), found in other teleosts (sea bream, fathead minnow, carp, Atlantic salmon and rainbow trout), shows significant differences compared to all other fish MHC class II β sequences and shares several features with classical and nonclassical tetrapod molecules^15^.

Among the over 30.000 teleost species, Antarctic notothenioid fishes could provide an interesting opportunity to study the evolution of the MHC system in a peculiar environment, where sea water reaches temperatures as low as −1.86 °C in the winter^18^. Low temperatures are thought to play a significant role in influencing the immune system, as they tend to inhibit immune responses and to modulate MHC genetic diversity^19–21^. As an example, low temperature has been hypothesized as a factor possibly leading to the loss of MHC II system in Gadiformes^22,23^. Antarctic notothenioids have developed key adaptations to cope with Antarctic extreme conditions, which are also involved in their pattern of adaptive radiation^24^. These include, among the others, anti-freeze glycoproteins, high mitochondrial density, cold-efficient microtubule assembly and physiological compensations for the lack of the swim bladder^25,26^. Moreover, Antarctic fish show peculiar aspects linked to their immune system, as evidenced by the unique features of their immunoglobulin genes compared to non-Antarctic species, such as the presence of a long hinge peptide^27,28^.

Within this framework, we decided to study the MHC class II β system in the icefish *Chionodraco hamatus*. Icefish belongs to Channichthyidae, the family that displays the most extreme adaptions to the Antarctic environment, which include the lack of haemoglobin due to the loss of globin genes^29^. This species is therefore a good candidate to investigate whether and in which measure the adaptation to low temperatures may have affected MCH II system organization and variability. To this end, we have identified two MHC class II β sequences in the gill transcriptome, and investigated their structural features and phylogenetic relationships. Furthermore, the genetic variability of the exon 2 region of both MHC II β genes was further examined using a deep targeted sequencing approach, and discussed in the light of the environmental and ecological forces acting on MHC II diversity.

## Results

### Analysis of MHC class II β sequences in *Chionodraco hamatus*

The two MHC class II β sequences identified from the *Chionodraco hamatus* gills transcriptome show about 41% amino acid identity and have been designated as Chha-DAB (accession number KY173352) and Chha-DBB (accession number KX398847) (see Figs S1 and S2 in supplementary files), respectively. The former comprised 747 bp and it encoded a 248 amino acid-long protein with a predicted 17 amino acid-long signal peptide, whereas the latter contained 759 bp and could be translated in 252 amino acids, with a predicted 21 amino acid-long signal peptide and one potential N-glycosylation site. The correctness of the two sequences was confirmed by cloning the nearly entire CDS from gill cDNA using the primers shown in Table 1.

**Table 1:** Primers used for MHC II β cloning, sequencing and expression analysis.

Gene	Primers Sequence 5′-3′ (forward, FW, and reverse, RV)	Accession number
MHC II β tot Chha-DAB (from cDNA)	GGCTTCTGTCCTCAGCGTCTGCC (FW) GTGGCTGGGAACCAGGATCCGTC (RV)	KY173352
MHC II β exon 2 Chha-DAB (from genomic DNA) inner amplification	tail1_FW *CAGGACCAGGGTACGGTG*CACGAGTAGCCGCTGTGAGTTC tail2_RV *CGCAGAGAGGCTCCGT*GGCAGGGCGTACTGGTATTCA	
MHC II β tot Chha-DBB (both from cDNA and genomic DNA)	GGGTATGAAGTTCTCGTTTTCACTG (FW) GAATTAGTTGGCACCAACTCTCGTCC (RV)	KX398847 KX886360
MHC II β exon 2 Chha-DBB (from genomic DNA) inner amplification	tail1_FW *CAGGACCAGGGTACGGT*GTCATGCTTTGTTCCACTGCCAG tail2_RV *CGCAGAGAGGCTCCGTG*CATTGCGATGTGGTTCTTGCATTTC	
Outer amplification (for both Chha-DAB and Chha-DBB)	A_tail1 CCATCTCATCCCTGCGTGTCTCCGACTCAG(X)_10_*CAGGACCAGGGTACGGTG* trP1_tail2 CCTCTCTATGGGCAGTCGGTGAT*CGCAGAGAGGCTCCGTG*	
MHC II β Chha-DAB exon 3	CACCCGGCCATGTTGGTCTGCAG (FW) GGCTGACGTGCTCCACCATGC (RV)	
MHC II β Chha-DBB exon 3	GCTCGTCTGCAGTGTGCACTAC (FW) GTGGGCGTGCTCCACCATG (RV)	
18S Ribosomal RNA	CCAACGAGCTGCTGACC (FW) CCGTTACCCGTGGTCC (RV)	AJ532569

Italicised letters correspond to the tail added to the exon 2 specific sequences.

**Figure 1:**
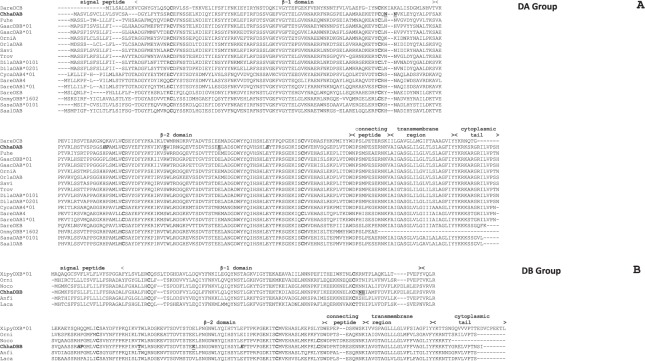
(**A**) Amino acid sequence alignment of the predicted icefish MHC II β Chha-DAB with selected DA group MHC sequences. The position of the cysteine residues found in the different sequences is shown in bold along the sequences. The amino acid residues potentially involved in binding with antigen peptides and CD4 are highlighted in bold and underlined, the ones potentially involved in interactions with MHC class II α chain are in bold and italics. Accession numbers: *Danio rerio* (zebrafish) AAA87894.1 (DareDCB); *Chionodraco hamatus* (icefish) KY173352 (ChhaDAB); *Fundulus heteroclitus* (mummichog) XP_012715816.2 (Fuhe); *Gasterosteus aculeatus* (stickleback) AAU01920.1 (GaacDBB*01); *Gasterosteus aculeatus* (stickleback) AAU01918.1 (GaacDAB*01); *Oreochromis niloticus* (Nile tilapia) XP_003459301.2 (OrniA); *Oryzias latipes* (medaka) BAA94279.1 (OrlaDAB); *Sander vitreus* (Walleye) AAO19848.1 (Savi); *Trachinotus ovatus* (pompano) APD68814.1 (Trov); *Dicentrarchus labrax* (sea bass) AM113466 (Dila-DAB*0101); *Dicentrarchus labrax* (sea bass) AM113467 (Dila-DAB*0201); *Cyprinus carpio* (common carp) CAA64709.1 (CycaDAB4*01); *Danio rerio* (zebrafish) AAA87891.1 (DareDAB4); *Danio rerio* (zebrafish) AAA50043.1 (DareDAB1*01); *Danio rerio* (zebrafish) AAA87895.1 (DareDEB); *Oncorhynchus mykiss* (rainbow trout) AF115531.1 (OnmyDBB*1602); *Salmo salar* (Atlantic salmon) CAA49725.1 (SasaDAB*0101); *Salvelinus alpinus* (Arctic char) ACI05078.1 (SalaDAB). (**B**) Amino acid sequence alignment of the predicted icefish MHC II β Chha-DBB with selected DB group MHC sequences. Accession numbers: *Xiphophorus pygmaeus* (Pigmy swordtail) AAS55044.1 (XipyDXB*01); *Oreochromis niloticus* (Nile tilapia) XP_019220627.1 (Orni); *Notothenia coriiceps* (Black rockcod) XP_010785298.1 (Noco); *Chionodraco hamatus* (icefish) KX398847 (ChhaDBB); *Anoplopoma fimbria* (sablefish) ACQ58371.1 (Anfi); *Lates calcarifer* (Asian sea bass) XP_018541680.1 (Laca).

Two multiple sequence alignments were produced using the icefish MHC class II β protein sequences inferred and selected homologous sequences found in the NCBI databases. The Chha-DAB sequence closely matched with sequences from the A lineage^7^ (Fig. 1, Panel A), displaying conservation of amino acid residues important for the functional activity of MHC. The β-1 domain comprised about 90 amino acid residues and was the least conserved region among the considered fish species. The β-2 domain, slightly larger than the β1-domain, started with an alanine or valine residue and ended with a conserved tryptophan residue. This domain showed more than 30% amino acid identity in all considered sequences. In addition, the transmembrane region showed a high amino acid identity, whereas the cytoplasmic tail exhibited inter-specific length variability, in spite of the presence of a tyrosine residue conserved at the beginning of all sequences. The cysteine residues found in the β-1 and β-2 domains, conserved in all species, are expected to be involved in the formation of two disulphide intra-chain bonds, as it happens in humans^30^. In classical mammalian MHC II molecules, two residues of the β-1 domain (H81 and N82) are involved in the formation of hydrogen bonds with the backbone of antigen peptide ligands^7,31,32^. While the latter was conserved in the Chha DAB sequence, the histidine residue was replaced by an asparagine (see Fig. 1, Panel A). Two other residues, located in the β-2 domain and fundamental in mammalian species for the binding with CD4 (S144 and E162), are conserved in the icefish sequence^7,33^.

The Chha-DBB sequence could be best aligned with sequences from the B lineage (Fig. 1, Panel B) and, therefore, it should be included in the nonclassical MHC class II β group. Similarly to Chha-DAB, the β-2 domain and the transmembrane region were highly conserved in all species. The residues of the β-1 domain that, in mammals, are involved in the formation of the backbone of antigen peptide ligands^7,31,32^ were not conserved, as we found an asparagine residue in position 81 and an histidine in position 82. The two other residues that form bonds with CD4 in mammals were also not conserved, as they were replaced by a threonine in position 144 and a lysine in position 162^7^.

**Figure 2:**
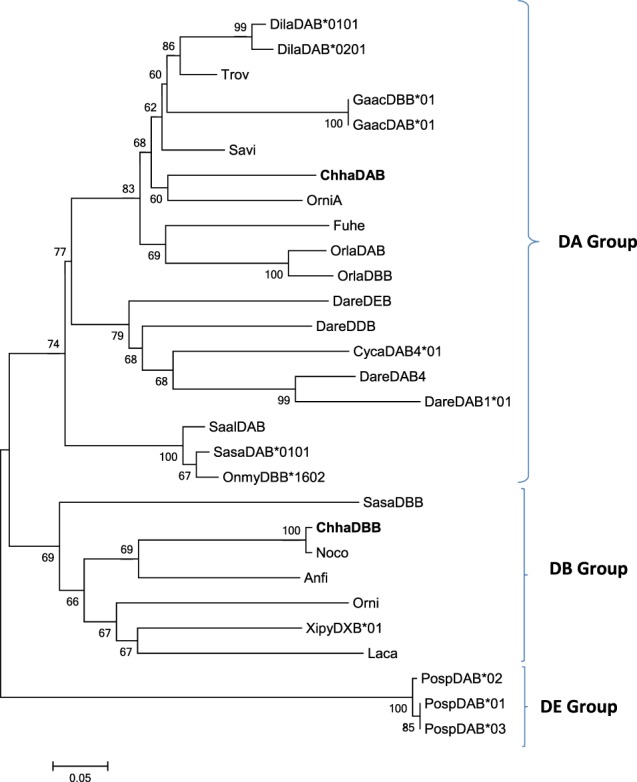
Phylogenetic tree analysis of icefish MHC II β sequences with selected fish MHC sequences. The phylogenetic tree was constructed using amino acid multiple alignments and the Neighbour-Joining method within the MEGA7 program. The percentage of replicate trees in which the associated taxa clustered together in the bootstrap test (10,000 replicates) was shown next to the branches. 0.1 indicates the genetic distance. Accession numbers are the same as reported in Fig. 1, with the exception of: *Oryzias latipes* (medaka) BAA94280.1 (OrlaDBB); *Danio rerio* (zebrafish) AAA87893.1 (DareDDB); *Polyodon spathula* (Mississippi paddlefish) ACZ26346.1 (PospDAB*01); *Polyodon spathula* (Mississippi paddlefish) ACZ26347.1 (PospDAB*02); *Polyodon spathula* (Mississippi paddlefish) ACZ26348.1 (PospDAB*03); *Salmo salar* (Atlantic salmon) ABX44766 (SasaDBB).

The phylogenetic analysis (Fig. 2) confirmed that the Chha-DAB sequence clustered with sequences of the A lineage and ChhaDBB with sequences from the B lineage.

**Figure 3:**
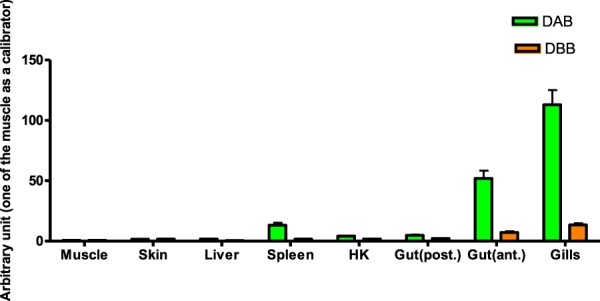
Expression levels of ChhaDAB and ChhaDBB in different organs and tissues.

The exon-intron organization of the Chha-DBB sequence (Supplementary Fig. S3) was studied using the primers shown in Table 1. Five exons were identified, with the connecting peptide and the transmembrane region being encoded by the same exon (exon 4). The basal expression analysis (Fig. 3) revealed that Chha-DAB was consistently expressed at higher levels compared to Chha-DBB, in all analysed tissues. In detail, the Chha-DAB mRNA was strongly expressed in gills and anterior gut, followed by lower levels in spleen, head kidney and posterior gut. Chha-DBB was also primarily produced by gills and anterior gut, albeit at levels 10 fold lower than Chha-DAB, and was found at very low levels in all other tissues.

### Analysis of MHC class II β exon 2 in *Chionodraco hamatus*

Overall, the amplicon analysis revealed a very low level of sequence diversity for the Chha-DBB exon 2 sequence. Indeed, a maximum of two variants per individual were detected by AMPLISAS, with an average of 1.11 variants per individual, estimated over the 44 samples that could be successfully analysed with Ion Torrent deep sequencing and the 10 samples re-analysed by Sanger sequencing. The translation of nucleotide sequences revealed that, in all cases, differences corresponded to synonymous substitutions, which therefore had no effect on the encoded amino acidic sequence.

**Figure 4:**
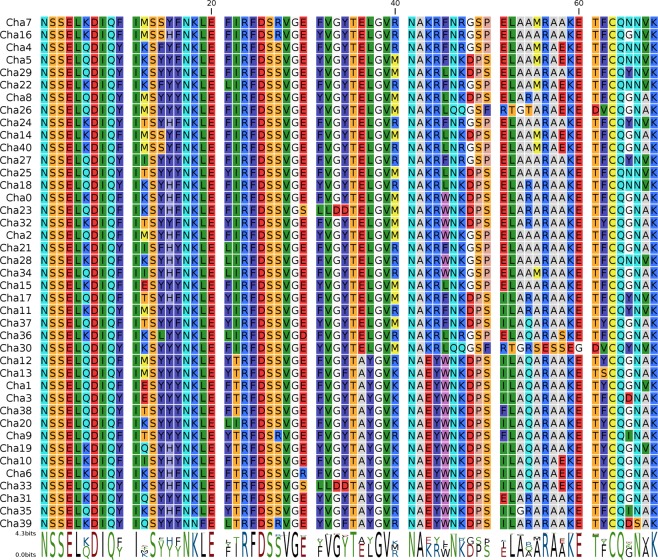
Multiple sequence alignment of the 41 translated non redundant DAB amino acid sequences, based on strict clustering (sequences sharing less than 3 non-synonymous SNPs have been collapsed in a single consensus sequence). Sequence conservation is indicated below the multiple sequence alignment with a conservation logo.

**Figure 5:**
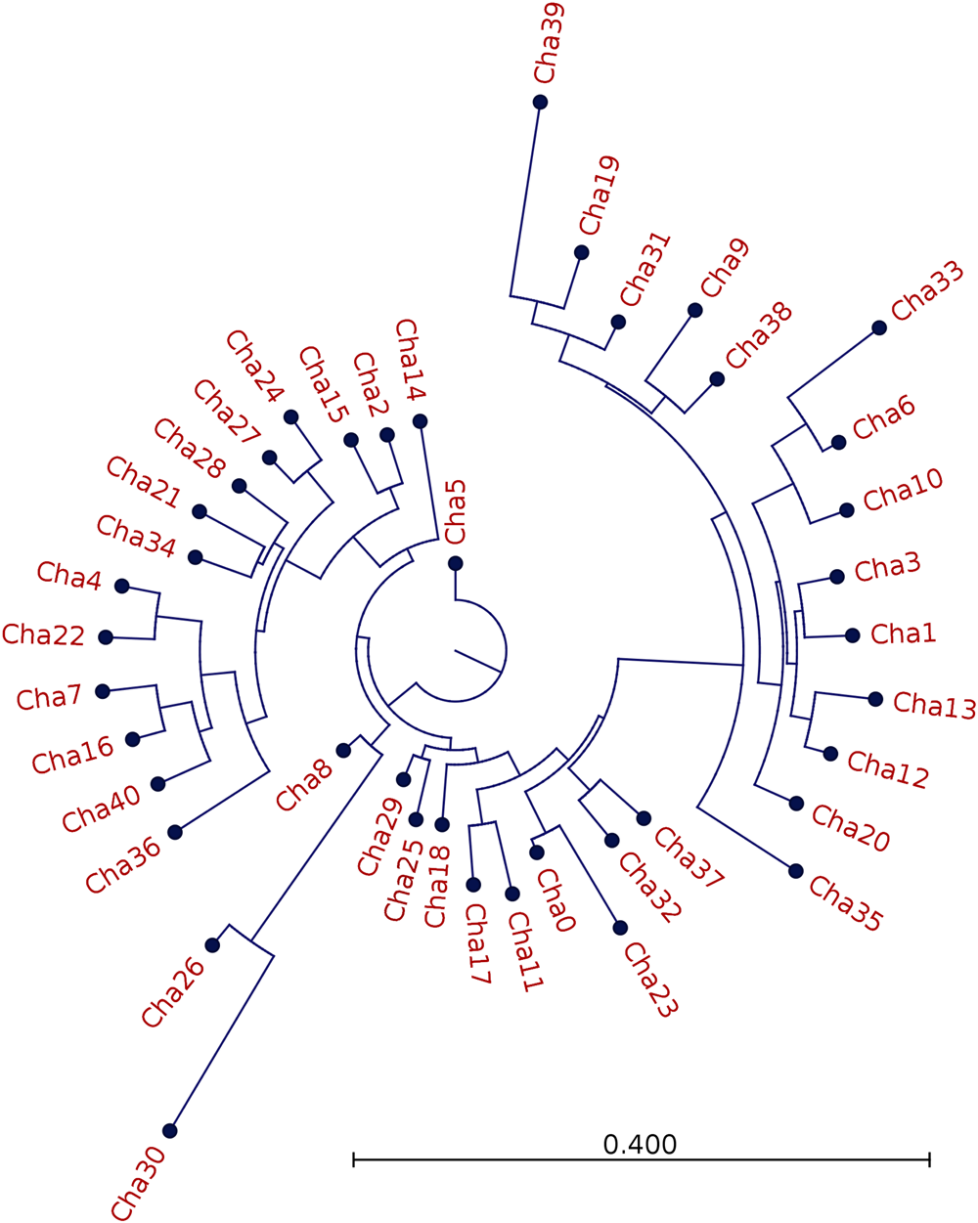
Circular phylogram depicting the relationships among the 41 non-redundant DAB translated peptides, inferred with Neighbour-Joining phylogenetic approach. For sequence numbering see Fig. 4.

**Figure 6:**
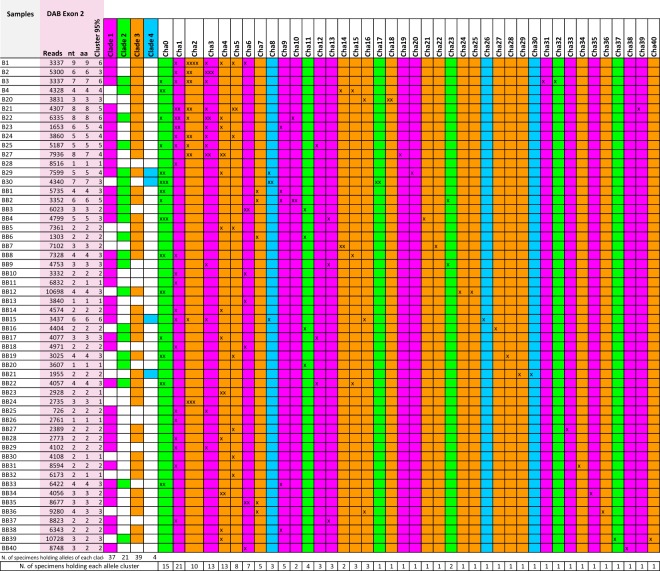
*Chionodraco hamatus* samples used for the MHC II β analyses with the indication of the corresponding number of reads from the sequencing, of identified peptides (aa), of putative nucleotide sequences (nt) and of clusters grouping sequences differing by less than 3 amino acids out of 68 (95%). The clade membership for each variant is also reported in different colours according to the phylogenetic analyses carried out: purple, clade 1; green, clade 2; orange, clade 3; blue, clade 4.

In stark contrast, the diversity of Chha-DAB exon 2 sequence was remarkably high, denoting an average number of 3.6 variants per individual, with estimates ranging from 1 (in B28, BB13, BB20, BB26) to 9 (in the B1 sample) at nucleotide sequence level. Overall, 92 different sequences were detected in the 54 investigated icefish. The diversity observed in Chha-DAB were largely translated at the protein level, determining the production of 87 unique amino acid sequences. To take into account the possibility of sequencing errors associated to the use of ION PGM platform (a combination of substitutions and indels)^34^, we adopted a highly conservative criterion for the estimate of sequence diversity, based on the clustering of sequences differing by less than 3 amino acids out of 68 (95% sequence identity threshold). This resulted in the identification of 41 distinct peptides, whose multiple sequence alignment is depicted in Fig. 4 and diversity is exemplified by the circular phylogram shown in Fig. 5. The clustering approach we adopted might have led to an underestimate of the MHC IIB exon 2 sequence diversity in icefish, due to the collapse of similar variants and recently diverged paralogs in a single consensus sequence. However, we argue that this strategy was appropriate for a preliminary, conservative survey (see Fig. 6). The 92 nucleotide sequences were also used to build a *p*-distance-based UPGMA dendrogram, which also recovered 41 clusters of genetically closely related sequences (see Fig. S4). Due to the lack of a reference genome for Channichthyidae, we are not presently able to assign these 92 putative variants at the corresponding loci. However, the presence of 9 different sequences in a single individual (B1) suggests that up to 5 loci are present. Moreover, the finding of very different numbers of variants in the various specimens analysed (1 to 9) suggests that copy-number variation (CNV) might occur.

**Figure 7:**
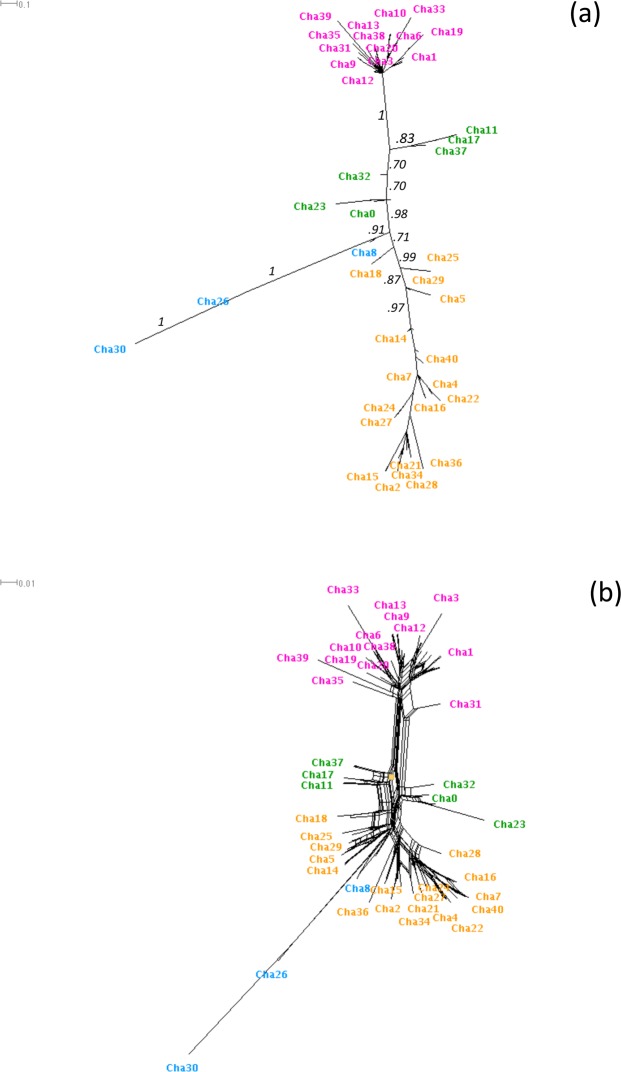
Phylogenetic relationships of exon 2 DAB variants according to (**a**) Bayesian inference and (**b**) Neighbor-Net network. Tips correspond to the 92 variants recorded but only the names of the 41 corresponding clusters are reported (Cha0 to Cha40). Posterior probabilities are reported on the branches. Different colours correspond to the four identified clades: purple, clade 1; green, clade 2; orange, clade 3; blue, clade 4.

The phylogenetic relationships among the 92 putative variants were investigated using Bayesian inference (BA), Maximum Likelihood (ML) and Neighbor-Net network (NN). Both BA and NN provided similar topologies showing four well-supported clades (Fig. 7), while ML recovered a slightly different and less supported topology, mainly due to the split of clade 3.

We consider the convergent estimate provided by BA/NN-network topology as the most reliable one, also taking into account that NN allows a better representation of the mixed genealogies often shown by MHC genes due to recombination and gene conversion^35^. The clades 1 and 3 included a comparable number of variants (14 and 18, respectively), while the clades 2 and 4 comprised a lower number of variants, i.e. 6 and 3, respectively (see Figs 6 and 7). The four clades were not evenly represented in the sampled specimens: variants belonging to clades 1 and 3 were recovered in the large majority of specimens (37 and 39, respectively), while those of clade 2 were just found in 21 out of 54 specimens, and only 4 individuals showed variants belonging to clade 3 (Fig. 6). The specimens with the higher number of variants often possessed those from three different clades, providing further evidence in support of the existence of multiple loci (Fig. 6). Nine animals only displayed variants belonging to a single clade, even though CNV was sometimes clearly detectable.

**Figure 8:**
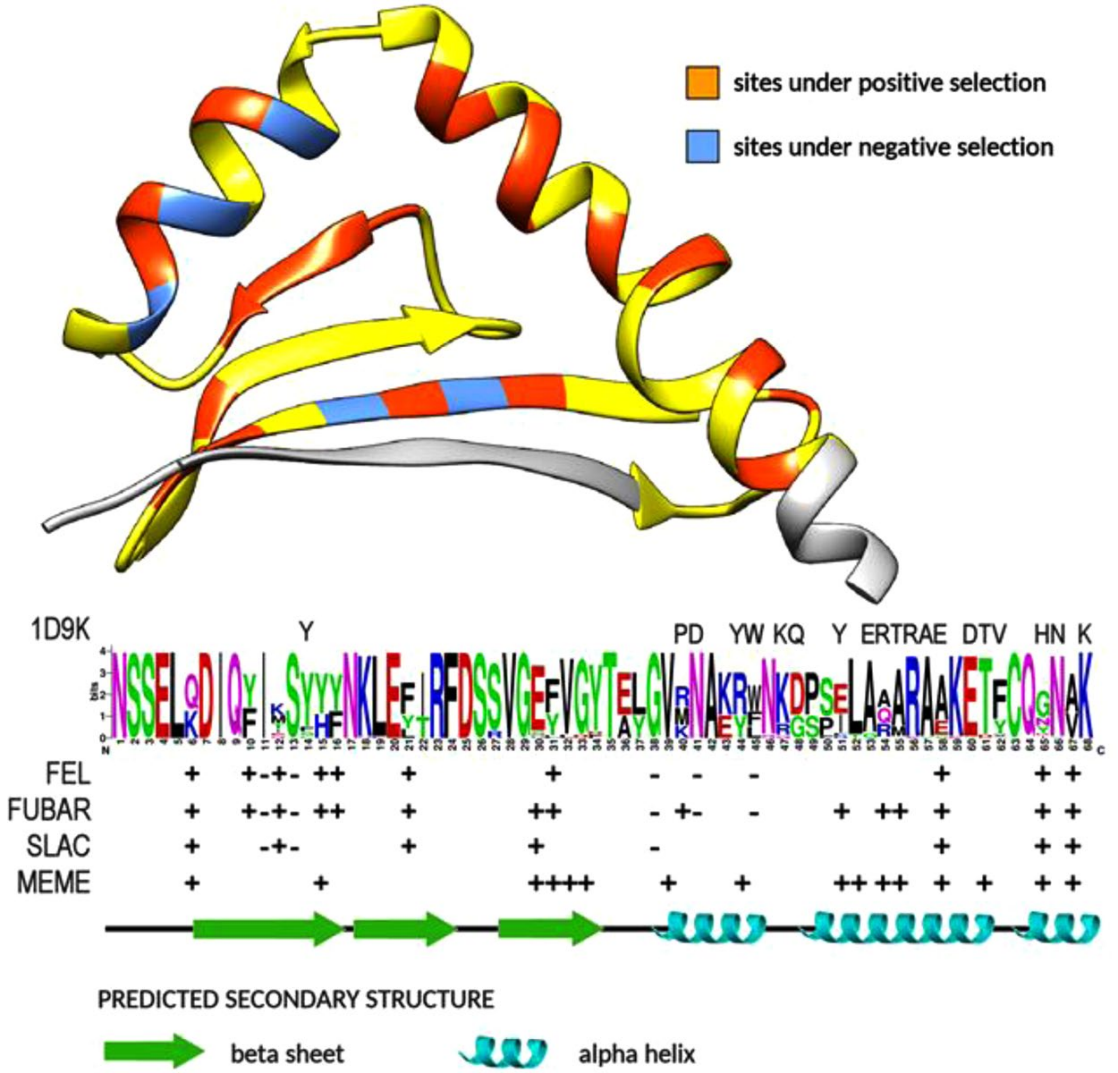
Structural modelling of the N-terminal domain of ChhaDAB sequence, based on the template crystal structure of the murine class II MHC I-Ab (PDB: 1MUJ). The sequence region targeted in this study is highlighted in yellow and the diversity of the observed peptides is indicated by a sequence conservation logo. Sites under positive and negative selection are indicated in orange and light blue, respectively, and detailed below the sequence logo with respect to FEL, FUBAR, SLAC (for pervasive selection) and MEME (for episodic selection). The expected secondary structure, inferred from the template structure, is indicated at the bottom of the Figure. 1D9K indicates amino acids known to be involved in the interaction with the antigen peptide and with the D10 T-cell α- and β-chains receptors in mouse.

The relative weight of selective forces acting on icefish Chha-DAB exon 2 sequence was evaluated based on the sequences encoding the aforementioned 41 peptide clusters. Despite some differences, the various tests were concordant in recognising multiple sites under pervasive positive selection (7 by SLAC, 10 by FEL and 15 by FUBAR) and 3 to 5 sites under significant purifying selection (Fig. 8). MEME further indicated the possibility that 16 out of the 68 codon positions analyzed (23.5%) might have undergone episodic positive selection.

The high predicted structural conservation of this domain between mouse and icefish allowed Phyre2 to model the structure of the icefish protein, sharing 40% sequence homology with the template structure, with 100% confidence and without gaps in the structural alignment. This permitted to localize with high confidence the sites under selective pressure on the three-dimensional structure of the N-terminal domain, as reported in Fig. 8. In detail, while sites predicted to be under pervasive positive selection were almost evenly distributed along the entire length of the sequence, the density of sites undergoing episodic selection was sensibly higher in the C-terminal portion of the α-helical region. The sites under negative selection were entirely located within the second of the four antiparallel β-sheets, interleaved with sites under strong positive selection and, in particular, in the N-terminal region of the α-helical domain.

## Discussion

The number of studies directed to explore the polymorphism of MHC genes has largely increased after the first reported evidence that, in vertebrates, the number of MHC variants affects crucial biological traits, such as pathogen immune recognition, mate choice and susceptibility to autoimmune diseases^36–38^. Among vertebrates, teleost fish count more than 30,000 species, which have undergone an extensive radiation in multiple lineages adapted to a great number of ecological niches, comprising extreme environments. An impressive phenotypic and genetic variation lies behind this taxonomic diversity, often underlying innovative evolutionary strategies including unconventional adaptive immune systems^39^. One of the most relevant ones is the case of the complete loss of the MHC II pathway genes in the entire order Gadiformes^23^. First documented in the Atlantic cod (*Gadus morhua*) and suggested to be connected to low temperature adaptation, this event has been subsequently demonstrated to be associated to an expansion of the MHC I repertoire^23,40^. However, the possible generalization of this pattern to other species adapted to cold environments remained unexplored.

Although widely studied for their peculiar adaptation to the extreme cold Antarctic environment, the MHC system of notothenioids has never been investigated in detail to date, and the presence of expressed sequences linked to this pathway has been only reported in a few transcriptomic analyses^41,42^. Our data allowed the identification of two different complete sequences from a gill transcriptome in the icefish *Chionodraco hamatus*, demonstrating the existence of two distinct MHC II lineages, classical and nonclassical, in this fish lineage. The genetic variation of the exon 2 region of the classical gene was also investigated, showing high sequence variability and copy number variation, coupled with signatures of selective pressure.

### Sequence analysis

Both sequence and phylogenetic analysis revealed that the two icefish MHC II transcripts belonged to two distinct lineages, i.e. the DA lineage (ChhaDAB, classical) and the DB lineage (ChhaDBB, nonclassical). Two out of the three amino acid residues proposed to be fundamental for the interactions with the MHC class II α chain in mammals were conserved in the two icefish sequences (His125 and Glu177 in the ChhaDAB sequence)^30^. On the other hand, the third residue (a histidine) was not present and, like in most of the teleosts sequences, it was substituted by a proline (Pro126) in the ChhaDAB sequence. The transmembrane domain, the most conserved region of the two molecules, shows the presence of many glycine residues in a structural motif that in mammals is suggested to be involved in the assembly of the MHC α and β complex^43^. Exon 2 encodes most of the β-1 domain of Chha-DAB, which includes in mammals several of the residues involved in the interaction with antigen peptide, as well as with the D10 T-cell antigen receptors α and β chains^32^. However, the comparison between fish and mammalian sequences revealed that only a few of these residues are evolutionarily conserved, as seen here for *Chionodraco hamatus* and also previously demonstrated in other teleosts^44^.

The ChhaDAB sequence was highly expressed in gills, a fundamental tissue of the fish immune system, due to its exposure to waterborne pathogens. This observation is in agreement with gene expression data previously reported in European sea bass and Atlantic salmon^23,44^. High expression levels were found also in the anterior gut, the section of the intestine accounting for the highest levels of MHC II β transcription in sea bass^45^, followed by spleen and head kidney. On the contrary, the expression of ChhaDBB transcripts could be detected mainly in gills and in anterior gut, at levels lower by 10 folds than ChhaDAB. These expression data perfectly matched those expected for MHC class II β genes comprised in the DA group (high expression in most tissues) and in the DB group (low tissue-specific expression), respectively^7,15^.

### Exon 2 variation

The exon 2 sequence of the ChhaDBB did not show any significant variation (i.e. the few SNPs identified were synonymous), similarly to what has been observed for all teleost nonclassical MHC class II genes analyzed until now, either from the DB or the DE group^7,15,16,46^. In stark contrast, the 92 putative variants identified for the ChhaDAB exon 2 encoded at least 41 different peptides. A matrix with pairwise differences and percentage of similarities, provided as Supplementary Fig. S5, enables to further appreciate the variability of these peptides, highlighting an average difference of 14.84 out of 68 amino acids. As previously mentioned, due to the lack of a reference genome, these variants could not be assigned to a specific locus, as in other studies involving non-model teleosts^39^. However, our data allowed identifying 1 to 9 variants per single individual, which suggests that up to 5 loci are present and that copy-number variation (CNV) occurs. These findings are in agreement with observations from other species, where up to ten MHC II B locus copies can be present^2–10^, with CNV between individuals belonging to the same population^15^. The phylogenetic analyses revealed that the variants observed belong to 4 distinct clades; two such clades were relatively large and diversified, while the other two only included six and three variants, respectively. The specimens with higher copy numbers often showed variants belonging to the three larger clades, further supporting that many loci are present. The reasons behind the lack of variants pertaining to the two smaller clades in several individuals are currently unknown, but we need to point out that since the approach targeted genomic DNA, some of the variants identified may correspond to pseudogenic copies with no biological relevance, hence more easily prone to accumulate mutations and quickly diverge from functional variants.

The number of DA-group MHC class II β alleles previously reported in different teleost species is quite variable and, although the methodology used may influence the results, a high heterogeneity emerged from the studies published so far. As an example, 22 and 42 alleles have been recorded in the IPD-MHC Database for the rainbow trout *OnmyDAB* locus and for the Atlantic salmon *SasaDAB* locus, respectively^7^. PCRs from genomic DNA resulted in the amplification of 13 distinct alleles of DAB genes in medaka, and 69 distinct alleles with up to 25 alleles per individual were found in Neotropical cichlids^17,47^. Moreover, 48 full-length functional MHC-DAB alleles have been identified in miiuy croaker by cDNA cloning^48^. In natural populations of European minnows 162 variants were isolated for the DAB1 gene and 177 for DAB3 using 454 sequencing, and 87 putative alleles with 1–6 alleles per individual have been identified in guppy by Illumina sequencing^12,49^. The level of sequence variation of ChhaDAB exon 2 in *Chionodraco hamatus* is high, especially considering the very strict thresholds we applied for clustering. Moreover, this study only targeted a single population, whereas other studies reporting similar sequence diversity in teleosts took into account many populations or even different species.

The finding of such a high genetic variation shows that the adaptation to cold does not necessarily determine a loss of variability or even a total withdrawal of the MHC II system, as hypothesized in the case of Gadiformes, and that, on the contrary, the genetic variation of MHC II may remain high even in an extremely cold environment^22^.

A possible enhancer of MHC genetic diversity is pathogen/parasite-mediated selection: disease-based models assume that co-evolutionary interactions between hosts and parasites result in balancing selection, which maximizes MHC polymorphisms^50^. Several studies have associated either the performance of single genotypes or overall MHC diversity with parasite infections. For example, in Atlantic salmon it has been evidenced that some haplotypes influence the resistance to the bacterial pathogen *Aeromonas salmonicida* and others are on the contrary significantly associated with susceptibility towards this disease^10,51,52^. In the European minnows the genetic diversity of the DAB3 gene seems to have been evolutionary derived mainly from host-pathogen interactions^49^. The host-parasite relationships might have led to MHC polymorphism through balancing selection: heterozygote advantage, negative frequency-dependent selection, or fluctuating selection pressures may act, also synergistically, to maintain MHC variation^5,37,53^. However, parasitic infection is also known to induce MHC class II expression, to elicit antibody production in the adaptive immune response. It has been shown that specific differential expression of MHC loci linked to polymorphism promoters are associated with disease in fish, thus making those parameters a direct target of natural selection^54^. In a natural population of three-spined stickleback (*Gasterosteus aculeatus*) the expression of MHC class II genes was positively correlated with parasitic load, which indicated increased immune activation of MHC when infection is frequent. A link between the polymorphism of coding sequences of MHC genes, which themselves regulates parasitic infection, and their expression level was observed^54^.

Although not directly taking into account the effects of parasite load, our data showed evidence of different mechanisms of selection in the ChhaDAB exon 2. The limitations due to the lack of assignment of variants to single loci prevented us from discussing in depth the location and types of selection. Nevertheless, taken altogether our data show that selective processes are relevant in determining genetic diversity of the immune system. This finding is in overall agreement with a previous study carried out on three species of the genus *Chionodraco* (including *Chionodraco hamatus*), which evidenced the presence of microsatellite outlier loci, thereby suggesting that selective pressures may have played a role in local adaptation, besides species divergence, in icefish^55^. Interestingly, one of these loci (Ch684, involved in the differentiation of *C. rastrospinosus*) was located at the 3′ region of a calmodulin gene that mediates many relevant cellular processes including, among the others, immune response.

Icefish show quite high level of parasitic load by different species of larval helminths^56,57^. Since parasites could exert strong selection pressures, and infection could be influenced by both variation of the MHC class II genes and their expression patterns, icefish would be of high interest to study these genes, their promoters and expression levels in differentially parasitized specimens.

## Conclusions

We have identified classical and nonclassical MHC II sequences in the Antarctic icefish *C. hamatus*, with the classical sequence being characterized by high molecular diversity. The results of a recent study have suggested that low temperature may have represented one of the major environmental factors underlying the development of a peculiar adaptive immune system in Atlantic cod, possibly influencing the loss of the MHC II in this species^22^. However, our finding of fully functional and highly diversified MHC class II β genes in icefish revealed that the adaptation to freezing environments cannot be regarded as a factor universally linked to the loss of MHC II genes.

On the contrary, we can hypothesize that pathogen-mediated selection may be involved in shaping the high sequence diversity of icefish classical MHC genes. Although the detection of the kind of selective mechanisms involved in such process can be puzzling in wild populations, the examination of MHC and neutral variation coupled with the determination of pathogen/parasite loads may represent a reliable approach to unravel this problem^5^. Altogether, the high diversity of the ChhaDAB exon 2, its heterogeneity among individuals and in expression levels, together with the signature of selective processes and the differential parasitic load in icefish individuals, make Antarctic fish-parasite systems an interesting case study.

## Materials and Methods

### RNA and DNA extraction

Adult *Chionodraco hamatus* specimens (mean weight 150 ± 10.5 grams) were collected with fishing lines and hooks at depths between 50 and 150 metres in Ross sea, Terra Nova Bay (74°38.485′; 164°03.726′) during the Italian XXXI and XXXII expeditions (years 2015/16, samples labelled B, and 2016/17, samples labelled BB, as listed in Fig. 6). Fish sampling was carried out under permission and following the Protocol on Environmental Protection to the Antarctic Treaty, Annex II, art. 3. Fish were maintained in 1000-litres plastic tanks with running marine water at −0.5 ± 1.2 °C in continuous dim light. The fish were killed by cervical concussion, and gills were immediately removed and disrupted by pushing tissue fragments through nylon cell strainers (100 μm) in ice-cold HBSS media (Gibco). The cell suspensions were centrifuged at 700 *g* at 4 °C for 10 min, the supernatant was discarded and cell pellets dissolved in one ml of Trisure (Bioline). The total RNA was isolated according to the manufacturer’s instructions and RNA concentration was determined using PicoDrop Microlitre Spectrophotometer Version 1.07. The RNA integrity was verified by staining of the 16 S ribosomal RNA bands on a denaturing 1% (w/v) agarose gel. For reverse transcription and cDNA production, the BioScript RNase H minus (Bioline) enzyme was used following the handbook protocol: in brief, 2 μg of total RNA was mixed with 1 μl of random hexamer (0.2 μg/μl, Amersham Pharmacia) and nuclease free water was added to a 12 μl final volume. The mixture was incubated at 70 °C for 5 min, and then cooled on ice. Subsequently, 0.4 μl of a reaction mix containing 100 mM dNTPs (25 mM each; Promega), 4 μl of 5X Reaction buffer, 0.25 μl of BioScript at 200 u/μl and nuclease free water to a 20 μl final volume were added. The solution was incubated at 25 °C for 10 min, then at 37 °C for 60 min. Finally, the reaction was stopped by heating at 70 °C for 10 min.

Genomic DNA was isolated from the muscle of 54 *C. hamatus* adults (see Fig. 6) using the Wizard Genomic DNA Purification kit (Promega) following manufacturer’s instructions. Genomic DNA was dissolved in water at a final concentration of 20 ngµl^−1^ after quantification with a PicoDrop Microlitre Spectrophotometer Version 1.07.

### Identification of two MHC class II β sequences from *Chionodraco hamatus* gills

Expressed MHC class II β sequences were recovered from a gill transcriptome of *C. hamatus*, generated as follows. Total RNA was extracted from the gill tissue of a single specimen of unspecified sex, weighting about 150 grams, using the same protocol described in the previous section. This material was used to prepare a sequencing library using an Illumina TruSeq kit (Illumina, San Diego, CA, USA), which was sequenced on a single lane of an Illumina HiSeq 2000 platform, with a 2 × 100 bp strategy. The sequencing output, accounting for 77 million reads (Bioproject: PRJNA343733), was *de novo* assembled with the CLC Genomics Workbench v.9 (Qiagen, Hilden, Germany), using default parameters (manuscript in preparation). The MHC class II β from sea bass (accession number AM113466) was used as a query for tBLASTn searches, to identify potential matches based on an e-value threshold of 1E^−5^. The identity of positive hits was assessed by reciprocal BLAST against the UniProtKB and nr protein sequence databases and the correct assembly of the sequences was evaluated by the inspection of read mapping distribution along the length of the transcripts.

To investigate the basal expression levels of the two MHC sequences, three icefish juveniles were sampled and different tissues (muscle, skin, liver, spleen, head kidney (HK), anterior gut (gut(ant.)), posterior gut (gut(post.)), gills) were obtained as described before for gills. Total RNA was isolated from each tissue separately with TRIsure (Bioline), resuspended in DEPC treated water and used for reverse-transcription real-time quantitative PCR in individual samples. For reverse transcription, the BioScript RNase H minus (Bioline) enzyme was used according to the manufacturer’s instructions. The expression level of the two MHC transcripts was determined with a Mx3000P real-time PCR system (Stratagene). Specific PCR primers were designed for the amplification of about 250 bp products from the two MHC sequences isoforms in the conserved exon 3 region (see Table 1). Relative quantitation was performed by comparing the levels of the target transcripts (the MHC sequences) to a reference transcript (calibrator, one of the tissue with the lowest MHC expression, in this case the muscle). A normalizer target (18S ribosomal RNA, see Table 1) was included to correct for differences in total cDNA input between samples. The results have been expressed as the mean ± SD of the results obtained from the three fish (two technical replicates were performed for each fish).

### Cloning and analyses of MHC class II β sequences

The occurrence of two MHC class II β expressed sequences identified by the *in silico* screening of the transcriptome has been further confirmed by the sequencing of the corresponding PCR amplicons obtained from icefish gills cDNA. The architecture of one of the two MHC class II β genes has been studied on a genomic level after sequencing from genomic DNA. The used primers are highlighted in Table 1.

The sequences were compared with counterparts in other species with the EMBOSS Pairwise Alignment tool. They were analysed for the presence of a signal peptide and of N-linked glycosylation sites using the softwares SignalP^58^ and NetNGlyc 1.0 Server, respectively. Multiple sequence alignment of icefish MHC class II β and selected homologous proteins from other species was carried out using CLUSTALW in the MEGA7 Software^59^. Neighbour-Joining (NJ) algorithm tree was constructed with MEGA7^59^ using the multiple sequence alignment as an input and the Poisson substitution model. The support of tree topology was evaluated by the generation of 10000 bootstrap replicates.

### NGS sequencing and analysis of MHC class II β sequences

For both MHC class II β sequences, a PCR system amplifying the gene region corresponding to exon 2 was designed. The amplicon size is 249 bp long for MHC class II β DAB and 224 bp for MHC class II β DBB. These amplicon sizes were compatible with massive parallel sequencing on an ION PGM instrument (LIFE technologies, Carlsbad, USA) of PCR products obtained from the genomic DNA of 54 animals (see Fig. 6).

In detail, a first amplification step (inner PCR) was carried out for each sample in a 25 µL reaction volume containing the Brilliant SYBR Green Q-PCR Master Mix, 0.25 µL primer forward modified at 5′ with tail1 (10 µM) (see Table. 1), 0.25 µL primer modified at 5′ with tail 2 (10 µM) (see Table 1), and 1 µL (20 ng) of each DNA template following manufacturer’s instructions. The PCR amplifications were performed in a Mx3000P^TM^ real-time PCR System (Stratagene) and the PCR conditions were: 95 °C for 10 min, followed by cycles of 95 °C for 45 s, 52 °C for 45 s and 72 °C for 45 s. For each processed sample, the number of cycles was adjusted in order to stop the reaction one cycle after the starting of the amplification (i.e. one cycle over the threshold value of the machine). The second PCR (outer PCR), that required the addition of barcodes and ION Torrent specific sequences to the amplicons, was performed using a forward primer containing (from 5′ to 3′) the A adaptor, a sample-specific 10 bp barcode and the tail1 of the initial PCR primers, and a reverse primer with (from 5′ to 3′) the trP2 adaptor sequence and the reverse tail. Information on primer sequences are reported in Table 1. The reaction was performed with a CFX 96™ PCR System (Bio-Rad, Hercules, USA) in a 25 µL volume containing 10 µL HotMasterMix 5Prime, 1,25 µL EvaGreen™ 20 × , 1,5 μl barcoded primer (10 µM), 1 μl of the first PCR product with the following conditions: 6–8 cycles of 94 °C for 10 s, 60 °C for 10 s, 65 °C for 40 s and a final extension of 72 °C for 3 min. The quality and size of the amplicons were assessed by agarose gel electrophoresis and PCR products were subsequently pooled together in equimolar amounts. The libraries were purified by the E-Gel® SizeSelect™ (Invitrogen, Carlsbad, USA) and the size and the amount of the products was verified with Agilent 2100 Bioanalyzer and Quibit Fluorometer (Thermo Fisher Scientific, Waltham, USA).

The libraries were submitted to emulsion PCR on the Ion OneTouch™ 2 system using the Ion PGM™ Template Hi-Q OT2 View (Life Technologies, USA) according to the manufacturer’s instructions. Ion sphere particles (ISP) were enriched using the E/S module. Resultant live ISPs were loaded and sequenced on an Ion 314 chip (Life Technologies). The quality check and subsequent analyses of raw reads were carried out with the CLC genomics workbench 9.5 using the following tools: “Demultiplex Reads” to bin reads to samples, “Trim reads” for quality and adaptors trimming, “Maps Reads to reference” for mapping reads to the two MHC class II β genes and “Basic variant detection” with Minimum coverage = 40, Minimum count = 20, Minimum frequency = 5% parameters for the identification of variants in the amplicon reads. For allele recognition and numbering, we took advantage of the software AMPLISAS^60^, specifically developed for amplicon sequencing analysis of MHC genes. This software is designed to filter out erroneous sequences and to perform unique sequence clustering. All the variants recognized by AMPLISAS were clustered with CD-HIT (http://weizhongli-lab.org/cdhit_suite/cgi-bin/index.cgi?cmd=cd-hit) at different sequence identity cut-off (0.98, 0.95 and 0.90). The representative proteins of the 0.95 identity clustering where aligned with Muscle^61^ and a cladistic tree was built with a NJ approach (Distance measure = Kimura Protein, Bootstrap = 1000 Replicates).

DBB sequence amplification was originally unsuccessful in 10 samples (B1, B2, B4, B20, B25, B28, BB2, BB4, BB7, BB29) which were therefore not subject to massive amplicon sequencing. In such cases, PCR was repeated; the 224 bp obtained amplicon was inserted into the p-GEM-T easy vector (Promega) and transfected into JM109 *Escherichia coli* competent cells (Promega). Plasmid DNA from ten independent clones from each sample was purified with the PureYield^TM^ Plasmid Miniprep System (Promega) and sequenced using the MWG DNA sequencing service.

The nucleotide sequences, which were aligned at translated amino acid sequence level, was used as an input for the detection of site-based pervasive selection analysis using the algorithms FEL (Fixed Effect Likelihood)^62^, FUBAR (Fast, Unconstrained Bayesian AppRoximation)^63^ and SLAC (Single-Likelihood Ancestor Counting)^62^. Episodic selection was evaluated with the MEME algorithm^64^. The aforementioned analyses were carried out using the Datamonkey adaptive evolution server^65^. The three-dimensional structure of Chha-DAB was modelled by homology with Phyre2^66^, using the experimentally determined structure of the murine class II MHC molecule I-A(b) (PDB ID: 1MUJ) as a template.

### Phylogenetic analysis of ChhaDAB exon 2

The genetic relationships among the 92 putative variants observed at the ChhaDAB exon 2 have been investigated by building an UPGMA dendrogram based on nucleotide *p*-distance under 1000 bootstrap replicates, as implemented in Mega 6.06^67^. This analysis confirmed the presence of 41 clusters of genetically similar putative variants, including 1 to 9 different nucleotide sequences (Fig. S4). The phylogenetic relationships among the variants were analysed using both Bayesian inference (BA) and Maximum Likelihood approaches (ML). The most appropriate nucleotide substitution model (GTR with a γ distribution) and the rates of base substitutions for each nucleotide pair were assessed using Mega 6.06 and used to set corresponding BA parameters in MrBayes^68^, running on the CIPRES portal. Two independent runs of four Markov chains Monte Carlo were run for 3 million generations and sampled every 1000 generations, with 25% fraction discarded as part of the burnin process. The ML analysis was carried out as implemented in Mega 6.06 using a GTR + G substitution model with 1000 bootstrap replicates. Both BA and ML trees were then visualized in SplitsTree v4.13, which was also used to build a Neighbor-Net network^69^.

### Use of experimental animals

All fishes were handled complying with the Guidelines of the European Union Council and of the Ethical Committee of the Tuscia University (Prof. Giuseppe Scapigliati, Prof. Nicola Lacetera, Prof. Nicla Romano) for the use of live animals. All experimental animal protocols were approved by the Ethical Committee of the Tuscia University (Prof. Giuseppe Scapigliati, Prof. Nicola Lacetera, Prof. Nicla Romano).

## Supplementary information

Supplementary files
