# Supplementary material for: Molecular and Structural Characterization of MHC Class II β Genes Reveals High Diversity in the Cold-Adapted Icefish Chionodraco hamatus
Source: Sci Rep. 2019 Apr 2;9:5523. doi: 10.1038/s41598-019-42003-5 (PMC6445107; doi:10.1038/s41598-019-42003-5)
Supplement: Supplementary file 1 — Supplementary files [file 41598_2019_42003_MOESM1_ESM.pdf]

MOLECULAR AND STRUCTURAL CHARACTERIZATION OF MHC CLASS II β GENES REVEALS HIGH DIVERSITY IN THE COLD-ADAPTED ICEFISH *Chionodraco hamatus*

Marco Gerdol[1°], Daniela Lucente[2,5°], Francesco Buonocore[3*], Elia Poerio[3], Giuseppe Scapigliati[3], Simonetta Mattiucci[4], Alberto Pallavicini[1,6], Roberta Cimmaruta[5]

[1]Department of Life Sciences, University of Trieste, Trieste, Italy

[2] University of Tuscia, Viterbo, Italy

[3]Department for Innovation in Biological, Agrofood and Forest Systems, University of Tuscia, Viterbo, Italy

[4]Department of Public Health and Infectious Diseases, Section of Parasitology, Sapienza University of Rome, Rome, Italy

[5]Department of Ecological and Biological Sciences, University of Tuscia, Viterbo, Italy

[6]National Institute of Oceanography and Applied Geophysics

°These two Authors contributed equally to the paper

*Corresponding author at: Department for Innovation in Biological, Agro-food and Forest systems, University of Tuscia, Largo dell'Università snc, 05100 Viterbo (VT), Italy, E-mail address: fbuono@unitus.it

```
atggcttctgtcctcagcgtctgcctgctcgtcctcagcgtctgctcagcagatggatat
 M  A  S  V  L  S  V  C  L  L  V  L  S  V  C  S  A  D  G  Y
ctgttttacacgagtagccgctgtgagttcaactcctctgagctgaaggacatccagtac
 L  F  Y  T  S  S  R  C  E  F  N  S  S  E  L  K  D  I  Q  Y
atcaagtcattctattacaacaagttggagttcatcaggttcgacagcagcgtgggggag
 I  K  S  F  Y  Y  N  K  L  E  F  I  R  F  D  S  S  V  G  E
tttgttggatacactgagctgggggtgaggaacgcaaagcgcttcaacaggggttctcca
 F  V  G  Y  T  E  L  G  V  R  N  A  K  R  F  N  R  G  S  P
gaactggccgcgatgagagcagagaaggagacgttctgccaaaacaacgttaagcttgaa
 E  L  A  A  M  R  A  E  K  E  T  F  C  Q  N  N  V  K  L  E
taccagtacgccctgcctttctcagcgaagccctacgtccggcttcactccacggtctcc
 Y  Q  Y  A  L  P  F  S  A  K  P  Y  V  R  L  H  S  T  V  S
cccagcggatcacacccggccatgttggtctgcagcgtctacgagttctaccccaaagtc
 P  S  G  S  H  P  A  M  L  V  C  S  V  Y  E  F  Y  P  K  V
atcaaagtgagctggatcaggaacggacaggaagtcacctctgatgtcacttcctctgaa
 I  K  V  S  W  I  R  N  G  Q  E  V  T  S  D  V  T  S  S  E
gagctggctgacagtgactggtactaccaggtccactcccacctggagtacacgcccagg
 E  L  A  D  S  D  W  Y  Y  Q  V  H  S  H  L  E  Y  T  P  R
tctggagacaagatctcctgcatggtggagcacgtcagccagggagagcctctggttact
 S  G  D  K  I  S  C  M  V  E  H  V  S  Q  G  E  P  L  V  T
gactgggacccctccatgccagagtctgagaggaacaaggtagccatcggagctgcagga
 D  W  D  P  S  M  P  E  S  E  R  N  K  V  A  I  G  A  A  G
ctgatcctgggtctgaccttatctctggccgggttcatctactacaagaggaagtcccga
 L  I  L  G  L  T  L  S  L  A  G  F  I  Y  Y  K  R  K  S  R
ggacggatcctggttcccagccactaa
 G  R  I  L  V  P  S  H  -
```

Figure S1.The nucleotide and amino acid sequence of icefish MHC class II β Chha DAB. Start and stop codons are highlighted in red and signal peptide is in green.

atgggtatgaagttctcgttttcactgctgtttctgatcctttttttttcaagagctgat
 M G M K F S F S L L F L I L F F S R A D
gctcttttttggtcatgctttgttccactgccagtttacttcccctgatgactttgtttat
 A L F G H A L F H C Q F T S P D D F V Y
ttgggacaacttttcttcaataaagtgctacaactccaatacaacagcactttagggaag
 L G Q L F F N K V L Q L Q Y N S T L G K
tataccggctacacagagaaaacgaaagatattgcagaaggcctcaacaaaaatccaaaa
 Y T G Y T E K T K D I A E G L N K N P K
tttataaaagaagaaaaaaaaaatgaactgaaatgcaagaaccacatcgcaatgtttttt
 F I K E E K K N E L K C K N H I A M F F
gatgtcttttaaaaccagacctccatttggagccctctgtcagggtgaggtcagtacaa
 D V F L K P D L H L E P S V R V R S V Q
gcagcgagcagtcgacacccaggcatgctcgtctgcagtgtgcactacttctttcccaaa
 A A S S R H P G M L V C S V H Y F F P K
ccaatccgagtgacttggctgaggaacggaaaggaggtgacatctgatgtgacgtccact
 P I R V T W L R N G K E V T S D V T S T
gagaaactgtccaatggggattggcattatcagatccactcctacctggagttcacacct
 E K L S N G D W H Y Q I H S Y L E F T P
gtacctggagagaaaatcacctgcatggtggagcacgcccacctcatgaagcccaagctt
 V P G E K I T C M V E H A H L M K P K L
tgcgagtgggatccgagaactgatcgagagtcagagaacaacaagattgctgtcgggaca
 C E W D P R T D R E S E N N K I A V G T
gcggggctgctgctgggtctggtgtttttttgttgctgggctgatttacttcaagaagaaa
 A G L L L G L V F F V A G L I Y F K K K
acttatggacgagagttggtgccaactaattctatttaa
 T Y G R E L V P T N S I -

Figure S2.The nucleotide and amino acid sequence of icefish MHC class II β Chha DBB. Start and stop codons are highlighted in red, signal peptide is in green, and the potential N-glycosylation site is underlined.

ATGGGTATGAAGTTCTCGTTTTCACTGCTGTTTCTGATCCTTTTTTTTTCAAGAGCTG Exon 1 58 bp (signal peptide)

GTGAGTTACATTAAAATAGCTGACATTTACTTTTCTAGTTGCTATAACATACTACATTACATCTGTGGCATTTTATTGGAGTGTGCAATGCTTTATTCTA
CATTTTAGTAATACTTGTTGGCTTTGTGTTACAG Intron 1 134 bp

ATGCTCTTTTTGGTCATGCTTTGTTCCACTGCCAGTTTACTTCCCCTGATGACTTTGTTTATTTGGGACAACTTTTCTTCAATAAAGTGCTACAACTCCA
ATACAACAGCACTTTAGGGAAGTATACCGGCTACACAGAGAAAACGAAAGATATTGCAGAAGGCCTCAACAAAAATCCAAAATTTATAAAAGAAGAAAAA
AAAAATGAACTGAAATGCAAGAACCACATCGCAATGTTTTTTGATGTCTTTTTAAAACCAG Exon 2 261 bp (β-1 domain)

GTGATTGTGTGTATTGAAACTCTGTTAATAATTTAAATTACATATTGCATAATTATTTCGTAATTATGGGGTTTAAGTAACAGACTTTTAATCATATTTC
TGTGAAAAGTGTGCATAGAAAATACTAAATATACCCACTGTAATATTTTTGTTTTAACCTAAATATATCCAGAGAATTACTTGTGAAAACATTGAAGAGT
TGTTGTTTCATGCCAATTTGTTTCGTCTATAG Intron 2 232 bp

ACCTCCATTTGGAGCCCTCTGTCAGGGTGAGGTCAGTACAAGCAGCGAGCAGTCGACACCCAGGCATGCTCGTCTGCAGTGTGCACTACTTCTTTCCCAA
ACCAATCCGAGTGACTTGGCTGAGGAACGGAAAGGAGGTGACATCTGATGTGACGTCCACTGAGAAACTGTCCAATGGGGATTGGCATTATCAGATCCAC
TCCTACCTGGAGTTCACACCTGTACCTGGAGAGAAAATCACCTGCATGGTGGAGCACGCCCACCTCATGAAGCCCAAGCTTTGCGAGTGGG
Exon 3 291 bp (β-2 domain)

GTGGGGTAGGAAAAGTGACCGCTTGTTTTGAGTTAAATGTTATCCAGCCACATTTGTATTGATTAGAGTTATAGAAATGTGTGCTAAATGTGTTTTCAG
Introne 3 99 bp

ATCCGAGAACTGATCGAGAGTCAGAGAACAACAAGATTGCTGTCGGGACAGCGGGGCTGCTGCTGGGTCTGGTGTTTTTTGTTGCTGGGCTGATTTACTT
CAAGAAGAAAACTTATG Exon 4 117 bp (connecting peptide + transmembrane region)

GTGAGAGACACATACACTTTACCAAATTCTACCATCTTATCTCAAGACTATCAAGACACAAACATACAGTCTGTCACAAACTGTCTGTTTTTTTTTGGTC
ACCCCCTGTGTTAGGCTACGTTTGTTTTTACTGCTTGTCCCTGTGTTTTCCCTCCTGTTTGATTACCTTTCTGTTTTGCCTTCCCTCTTGTGTCCTGTCC
TTGGGATTAGTCTTGCATGTATTTAAGGTCTGGTTTTTTGTCAATTGTTGTCAGATCCTGTTCATTGTTCAATGGTGAGGTTTGTTCTGTTTTTGTATTT
AGAGATTTTCAACTTATTTTTTTGTGTCTTACCCTGCTCAGCCTGATTACCTGAAATTAAAGTTTTTTTGCTGTTCAAACCTTGTGCGTCCTGCTTTTGG
GTTCACCTTGCTCTGCTTTCAATCGTGAGAGTCTGCTTTTGATGAAACTGTTTAAAGATTGTATCACTGTTGTCTGAAGTCATAAACTCTCTGTTAATAA
TTTTGTCAAAATATTCTTTAGATAACAAAAAGCAACACGACCAGTGATTTCTTTCTTAAAGATCAGTTAAAGTAGCATTTTAAGTTTTGACATGAGCCTC
TAAGGCTTTGCTTAAAACAACATTAGTGGGACTCAAACTCAACTACAAGTATGTGTGACTTTGTTTTTCTTTTTGTTTCTTATTCTAG
Intron 4 688 bp

GACGAGAGTTGGTGCCAACTAATTCTATTTAA Exon 5 32 bp (cytoplasmic tail)

Figure S3. The exon-intron organization of the Chha-DBB sequence. The size of each intron and exon is reported.

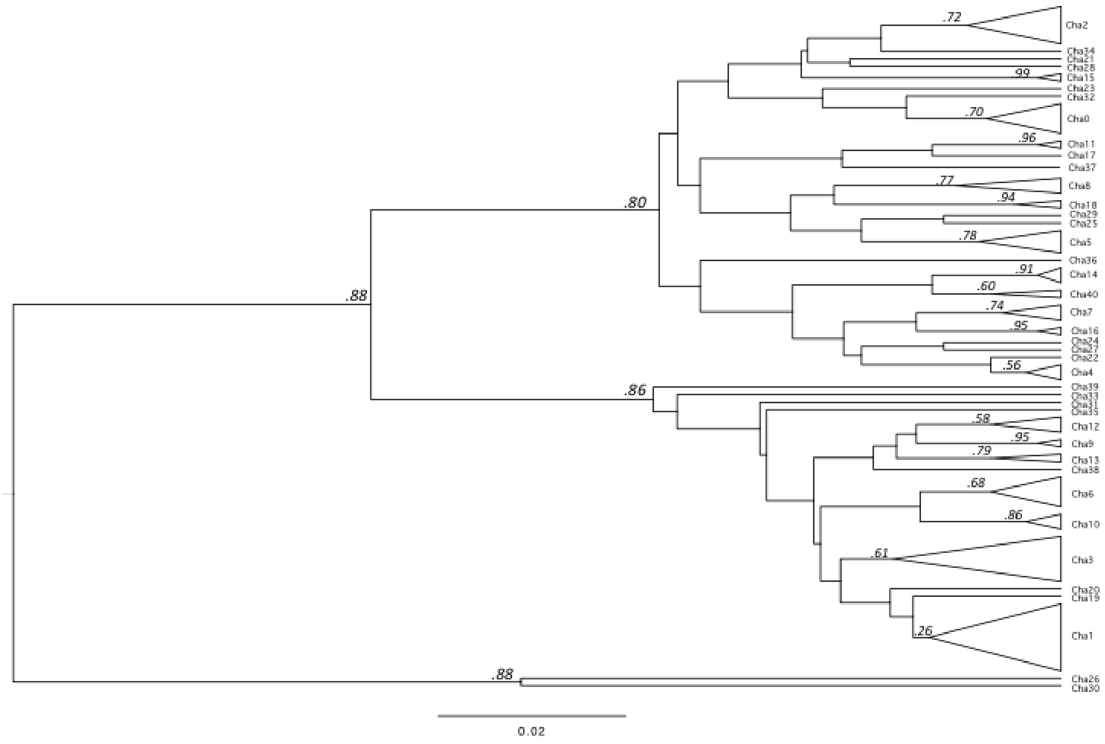

Figure S4. UPGMA dendrogram showing the genetic relationships among the 92 nucleotide sequences of DAB exon 2 recovered. Each triangle represent 2 to 9 genetically close sequences and labels correspond to the 41 peptide clusters obtained by grouping sequences differing by less than 3 aminoacids out of 68 (95%). Bootsrap values over 1000 replicates are reported at each relevant node. 0.02 represents the genetic distance.

	1	2	3	4	5	6	7	8	9	10	11	12	13	14	15	16	17	18	19	20	21	22	23	24	25	26	27	28	29	30	31	32	33	34	35	36	37	38	39	40	41
1		4	6	12	15	6	17	21	7	8	14	14	14	20	15	11	11	9	10	11	10	15	13	17	12	25	21	24	21	22	23	22	20	24	23	24	29	21	24	28	26
2	94,12		10	14	14	10	15	19	6	9	16	12	12	17	15	7	9	9	10	9	12	14	14	15	12	25	21	24	21	23	21	22	21	20	21	22	26	19	21	28	25
3	91,18	85,29		7	12	4	14	18	7	6	12	11	15	19	17	12	10	10	10	13	12	15	14	17	12	21	22	23	21	20	21	21	22	24	23	21	24	21	21	27	28
4	82,35	79,41	89,71		5	11	7	17	12	11	7	6	10	13	12	11	15	13	13	12	8	10	11	10	18	20	19	19	17	16	15	17	19	18	19	17	19	15	16	26	23
5	77,94	79,41	82,35	92,65		14	5	15	11	8	4	5	9	12	11	10	13	11	14	8	10	9	8	10	15	17	16	16	14	13	12	14	16	15	18	18	20	12	15	26	20
6	91,18	85,29	94,12	83,82	79,41		16	20	9	8	13	15	15	21	16	14	8	6	10	11	12	16	13	18	8	23	21	24	21	22	23	19	21	23	21	23	28	21	24	28	26
7	75,00	77,94	79,41	89,71	92,65	76,47		11	15	12	7	7	8	11	9	9	13	15	14	8	7	11	9	9	14	19	14	14	15	13	11	14	16	16	15	16	18	10	15	26	19
8	69,12	72,06	73,53	75,00	77,94	70,59	83,82		19	16	17	18	19	22	19	14	18	20	19	13	17	21	19	17	18	10	22	22	23	22	20	22	25	24	23	24	26	21	23	17	28
9	89,71	91,18	89,71	82,35	83,82	86,76	77,94	72,06		4	11	10	11	17	13	9	7	7	9	10	14	10	10	15	12	22	21	22	20	19	19	21	20	19	20	19	24	18	19	27	25
10	88,24	86,76	91,18	83,82	88,24	88,24	82,35	76,47	94,12		9	11	14	18	15	10	7	8	9	9	15	12	9	15	11	19	18	19	17	16	17	18	18	20	20	22	25	17	20	27	24
11	79,41	76,47	82,35	89,71	94,12	80,88	89,71	75,00	83,82	86,76		7	10	14	8	12	12	10	13	10	10	7	7	9	15	21	14	14	14	13	13	14	13	15	18	19	22	14	16	25	21
12	79,41	82,35	83,82	91,18	92,65	77,94	89,71	73,53	85,29	83,82	89,71		7	10	11	10	13	10	12	12	12	8	9	11	16	20	17	18	16	15	14	16	17	15	18	16	18	13	11	27	20
13	79,41	82,35	77,94	85,29	86,76	77,94	88,24	72,06	83,82	79,41	85,29	89,71		6	4	7	11	9	9	9	7	7	10	9	13	24	14	16	14	14	14	12	15	15	12	12	17	11	13	28	20
14	70,59	75,00	72,06	80,88	82,35	69,12	83,82	67,65	75,00	73,53	79,41	85,29	91,18		10	10	17	15	13	14	13	11	16	12	18	27	18	19	18	18	17	16	19	20	18	17	11	16	16	31	24
15	77,94	77,94	75,00	82,35	83,82	76,47	86,76	72,06	80,88	77,94	88,24	83,82	94,12	85,29		10	13	12	11	10	8	7	9	5	14	25	11	14	13	15	14	11	13	14	13	14	19	12	15	25	18
16	83,82	89,71	82,35	83,82	85,29	79,41	86,76	79,41	86,76	85,29	82,35	85,29	89,71	85,29	85,29		9	8	5	5	10	11	15	10	13	22	16	18	17	17	15	16	18	18	17	18	20	15	16	24	24
17	83,82	86,76	85,29	77,94	80,88	88,24	80,88	73,53	89,71	89,71	82,35	80,88	83,82	75,00	80,88	86,76		6	6	8	13	14	11	15	9	25	18	19	20	18	18	17	19	18	17	19	24	16	19	30	22
18	86,76	86,76	85,29	80,88	83,82	91,18	77,94	70,59	89,71	88,24	85,29	85,29	86,76	77,94	82,35	88,24	91,18		6	8	14	12	11	16	9	23	17	20	17	18	19	15	17	17	17	19	24	17	18	28	22
19	85,29	85,29	85,29	80,88	79,41	85,29	79,41	72,06	86,76	86,76	80,88	82,35	86,76	80,88	83,82	92,65	91,18	91,18		9	11	12	15	13	11	25	16	19	18	18	18	15	17	20	17	19	22	18	17	26	24
20	83,82	86,76	80,88	82,35	88,24	83,82	88,24	80,88	85,29	86,76	85,29	82,35	86,76	79,41	85,29	92,65	88,24	88,24	86,76		9	14	13	11	9	20	17	19	16	16	15	16	18	18	17	20	24	13	20	24	22
21	85,29	82,35	82,35	88,24	85,29	82,35	89,71	75,00	79,41	77,94	85,29	82,35	89,71	80,88	88,24	85,29	80,88	79,41	83,82	86,76		12	12	11	13	25	16	18	17	17	17	16	18	20	15	16	21	15	20	27	24
22	77,94	79,41	77,94	85,29	86,76	76,47	83,82	69,12	85,29	82,35	89,71	88,24	89,71	83,82	89,71	83,82	79,41	82,35	82,35	79,41	82,35		6	6	18	23	14	16	14	15	16	14	14	15	16	17	20	17	13	25	22
23	80,88	79,41	79,41	83,82	88,24	80,88	86,76	72,06	85,29	86,76	89,71	86,76	85,29	76,47	86,76	77,94	83,82	83,82	77,94	80,88	82,35	91,18		10	15	22	12	13	14	13	15	14	14	13	16	17	22	13	14	29	17
24	75,00	77,94	75,00	85,29	85,29	73,53	86,76	75,00	77,94	77,94	86,76	83,82	86,76	82,35	92,65	85,29	77,94	76,47	80,88	83,82	83,82	91,18	85,29		16	24	10	13	12	14	12	10	13	13	14	15	17	15	14	23	19
25	82,35	82,35	82,35	73,53	77,94	88,24	79,41	73,53	82,35	83,82	77,94	76,47	80,88	73,53	79,41	80,88	86,76	86,76	83,82	86,76	80,88	73,53	77,94	76,47		23	18	21	20	20	20	16	19	22	19	20	25	19	23	27	25
26	63,24	63,24	69,12	70,59	75,00	66,18	72,06	85,29	67,65	72,06	69,12	70,59	64,71	60,29	63,24	67,65	63,24	66,18	63,24	70,59	63,24	66,18	67,65	64,71	66,18		30	30	28	28	27	28	31	29	31	31	33	27	27	13	33
27	69,12	69,12	67,65	72,06	76,47	69,12	79,41	67,65	69,12	73,53	79,41	75,00	79,41	73,53	83,82	76,47	73,53	75,00	76,47	75,00	76,47	79,41	82,35	85,29	73,53	55,88		4	4	6	7	4	5	7	6	9	12	9	9	32	12
28	64,71	64,71	66,18	72,06	76,47	64,71	79,41	67,65	67,65	72,06	79,41	73,53	76,47	72,06	79,41	73,53	72,06	70,59	72,06	72,06	73,53	76,47	80,88	80,88	69,12	55,88	94,12		6	5	6	6	8	7	8	9	11	8	10	34	12
29	69,12	69,12	69,12	75,00	79,41	69,12	77,94	66,18	70,59	75,00	79,41	76,47	79,41	73,53	80,88	75,00	70,59	75,00	73,53	76,47	75,00	79,41	79,41	82,35	70,59	58,82	94,12	91,18		4	6	4	8	6	5	8	11	8	12	33	14
30	67,65	66,18	70,59	76,47	80,88	67,65	80,88	67,65	72,06	76,47	80,88	77,94	79,41	73,53	77,94	75,00	73,53	73,53	73,53	76,47	75,00	77,94	80,88	79,41	70,59	58,82	91,18	92,65	94,12		4	6	7	8	7	8	11	6	9	34	12
31	66,18	69,12	69,12	77,94	82,35	66,18	83,82	70,59	72,06	75,00	80,88	79,41	79,41	75,00	79,41	77,94	73,53	72,06	73,53	77,94	75,00	76,47	77,94	82,35	70,59	60,29	89,71	91,18	91,18	94,12		7	6	7	8	9	11	4	9	32	13
32	67,65	67,65	69,12	75,00	79,41	72,06	79,41	67,65	69,12	73,53	79,41	76,47	82,35	76,47	83,82	76,47	75,00	77,94	77,94	76,47	76,47	79,41	79,41	85,29	76,47	58,82	94,12	91,18	94,12	91,18	89,71		8	8	6	8	11	9	11	32	13
33	70,59	69,12	67,65	72,06	76,47	69,12	76,47	63,24	70,59	73,53	80,88	75,00	77,94	72,06	80,88	73,53	72,06	75,00	75,00	73,53	73,53	79,41	79,41	80,88	72,06	54,41	92,65	88,24	88,24	89,71	91,18	88,24		11	10	13	16	10	9	32	13
34	64,71	70,59	64,71	73,53	77,94	66,18	76,47	64,71	72,06	70,59	77,94	77,94	77,94	70,59	79,41	73,53	73,53	75,00	70,59	73,53	70,59	77,94	80,88	80,88	67,65	57,35	89,71	89,71	91,18	88,24	89,71	88,24	83,82		6	7	11	6	9	34	11
35	66,18	69,12	66,18	72,06	73,53	69,12	77,94	66,18	70,59	70,59	73,53	73,53	82,35	73,53	80,88	75,00	75,00	75,00	75,00	75,00	77,94	76,47	76,47	79,41	72,06	54,41	91,18	88,24	92,65	89,71	88,24	91,18	85,29	91,18		4	9	8	12	35	14
36	64,71	67,65	69,12	75,00	73,53	66,18	76,47	64,71	72,06	67,65	72,06	76,47	82,35	75,00	79,41	73,53	72,06	72,06	72,06	70,59	76,47	75,00	75,00	77,94	70,59	54,41	86,76	86,76	88,24	88,24	86,76	88,24	80,88	89,71	94,12		6	9	10	36	15
37	57,35	61,76	64,71	72,06	70,59	58,82	73,53	61,76	64,71	63,24	67,65	73,53	75,00	83,82	72,06	70,59	64,71	64,71	67,65	64,71	69,12	70,59	67,65	75,00	63,24	51,47	82,35	83,82	83,82	83,82	83,82	83,82	76,47	83,82	86,76	91,18		13	12	38	18
38	69,12	72,06	69,12	77,94	82,35	69,12	85,29	69,12	73,53	75,00	79,41	80,88	83,82	76,47	82,35	77,94	76,47	75,00	73,53	80,88	77,94	75,00	80,88	77,94	72,06	60,29	86,76	88,24	88,24	91,18	94,12	86,76	85,29	91,18	88,24	86,76	80,88		9	34	10
39	64,71	69,12	69,12	76,47	77,94	64,71	77,94	66,18	72,06	70,59	76,47	83,82	80,88	76,47	77,94	76,47	72,06	73,53	75,00	70,59	70,59	80,88	79,41	79,41	66,18	60,29	86,76	85,29	82,35	86,76	86,76	83,82	86,76	86,76	82,35	85,29	82,35	86,76		31	13
40	58,82	58,82	60,29	61,76	61,76	58,82	61,76	75,00	60,29	60,29	63,24	60,29	58,82	54,41	63,24	64,71	55,88	58,82	61,76	64,71	60,29	63,24	57,35	66,18	60,29	80,88	52,94	50,00	51,47	50,00	52,94	52,94	52,94	50,00	48,53	47,06	44,12	50,00	54,41		38
41	61,76	63,24	58,82	66,18	70,59	61,76	72,06	58,82	63,24	64,71	69,12	70,59	70,59	64,71	73,53	64,71	67,65	67,65	64,71	67,65	64,71	67,65	75,00	72,06	63,24	51,47	82,35	82,35	79,41	82,35	80,88	80,88	80,88	83,82	79,41	77,94	73,53	85,29	80,88	44,12	

Figure S5. Pairwise comparison showing the divergence among the 41 aminoacid sequences of DAB exon 2. The number of amino acid differences and the percentage of similarities per sequence from between sequences of the 41 highly divergent peptides is shown. The overall average differences is 14.84 out of a total length of 68 aminoacids
